# Deep Learning-Based Approach for Emotion Recognition Using Electroencephalography (EEG) Signals Using Bi-Directional Long Short-Term Memory (Bi-LSTM)

**DOI:** 10.3390/s22082976

**Published:** 2022-04-13

**Authors:** Mona Algarni, Faisal Saeed, Tawfik Al-Hadhrami, Fahad Ghabban, Mohammed Al-Sarem

**Affiliations:** 1 College of Computer Science and Engineering, Taibah University, Medina 41477, Saudi Arabia; fsaeed@taibahu.edu.sa (F.S.); fghaban@taibahu.edu.sa (F.G.); msarem@taibahu.edu.sa (M.A.-S.); 2 Computer Science and Artificial Intelligence Department, College of Computer and Cyber Sciences, University of Prince Mugrin, Medina 42241, Saudi Arabia; 3 DAAI Research Group, Department of Computing and Data Science, School of Computing and Digital Technology, Birmingham City University, Birmingham B4 7XG, UK; 4 School of Science and Technology, Nottingham Trent University, Nottingham NG11 8NS, UK; tawfik.al-hadhrami@ntu.ac.uk

**Keywords:** bi-directional long short-term memory, binary grey wolf optimizer, brain–computer interface, electroencephalography, emotion recognition

## Abstract

Emotions are an essential part of daily human communication. The emotional states and dynamics of the brain can be linked by electroencephalography (EEG) signals that can be used by the Brain–Computer Interface (BCI), to provide better human–machine interactions. Several studies have been conducted in the field of emotion recognition. However, one of the most important issues facing the emotion recognition process, using EEG signals, is the accuracy of recognition. This paper proposes a deep learning-based approach for emotion recognition through EEG signals, which includes data selection, feature extraction, feature selection and classification phases. This research serves the medical field, as the emotion recognition model helps diagnose psychological and behavioral disorders. The research contributes to improving the performance of the emotion recognition model to obtain more accurate results, which, in turn, aids in making the correct medical decisions. A standard pre-processed Database of Emotion Analysis using Physiological signaling (DEAP) was used in this work. The statistical features, wavelet features, and Hurst exponent were extracted from the dataset. The feature selection task was implemented through the Binary Gray Wolf Optimizer. At the classification stage, the stacked bi-directional Long Short-Term Memory (Bi-LSTM) Model was used to recognize human emotions. In this paper, emotions are classified into three main classes: arousal, valence and liking. The proposed approach achieved high accuracy compared to the methods used in past studies, with an average accuracy of 99.45%, 96.87% and 99.68% of valence, arousal, and liking, respectively, which is considered a high performance for the emotion recognition model.

## 1. Introduction

The Brain–Computer Interface (BCI) is a popular research topic in the field of health informatics. Its applications involve the analysis of electroencephalography signals (EEG) from the brain. Popular BCI applications include monitoring the health and abnormal activity of the brain, such as psychological seizures and detecting emotions. Emotion detection techniques help to detect the activity of mentally challenged people, who cannot explain their emotions.

Brain–Computer Interface (BCI) technology enables interaction between the brain and the computer and is one of the significant branches of Human–Computer Interaction (HCI). It is also considered one of the most essential modern research fields related to machine and deep learning and robotics. 

BCI technology works through sequential steps that aim to recognize the signals of the human brain and convert them into actions. After the signals are collected, they are processed according to the frequency, and time features are extracted; finally, the signals are classified. The results are converted into commands for the different devices, according to the application used. 

BCI actively contributes to helping patients find solutions to their health problems and improve the quality of healthy life for patients with motor disabilities or with various mental diseases [1], to help patients communicate with others or control their prosthetic limbs by determining the brain’s activities [2]. A study by Teles et al. confirmed that BCI-based devices can transmit and receive signals from the brain to control external devices, such as wheelchairs, and collect information about the user’s intentions [2].

In another study, the authors demonstrated that the electroencephalogram (EEG) signal has a vital role in diagnosing epileptic seizures [3]. The study also used the BCI technique to develop an emotion recognition model. In addition, BCI technology has been employed for non-medical uses, such as education and recreational games [4,5].

BCI techniques begin by collecting brain signals according to the purpose for which the signals are collected. This is done through various techniques, including EEG, functional magnetic resonance imaging (fMRI), and magnetoencephalograms (MEGs). The medical devices used to measure physiological signals differ in their accuracy, signal quality, length and measurement of the resulting frequencies. Physicians decide the appropriate devices for each patient’s case and how to collect the signals in the correct way, in cooperation with specialized medical technicians [6].

Deep learning (DL) has demonstrated tremendous capabilities in medical decision-making systems, including: wearable technology, image-processing applications, natural language processing. All of these aim to improve the quality of health care. DL algorithms are effective to support decision making when the inputs are quantifiable.

In the study by Kamnitsas et al. [7], the authors used MRI brain imaging to detect brain tumors and stroke etiology through 3D CNN. Abd-Ellah et al. [8] used three different CNN networks, AlexNets, VGG-16 and VGG-19, for the detection of cancerous tumors, while Deniz et al. [9] assessed bone fracture risk, using U-Nets from MR images.

Wearable technology constitutes one of the important applications of decision support in the field of health services, where vital data are collected by sensor units. In the study by [10], a smartphone was used as a sensor to measure, say, the output/data collected from accelerometers and gyroscopes to study human activity. The application used SIFT (scale-invariant feature transform) to extract the various features collected from smartphone sensors and then passed these features through a convolutional neural network to classify the signal and take the decision about a person’s health status.

In another study [11], the authors developed a decision support system that monitors mental health symptoms with wearable technology. The model collects facial expressions from the phone’s camera, speech from the phone’s microphone, and movement data through GPS, an accelerometer and gyroscopes. Using a smart watch, the electrical activity of the skin is monitored, and the model also measures social interaction through social networking.

The medical field needs a system to diagnose psychological and behavioral diseases. Psychological diseases are currently diagnosed using traditional methods, by asking the patients some questions or monitoring their behavior, which is, however, time consuming. The diagnosis may also not be accurate, because it depends on the patient’s responses to the questions. Many studies have also used external emotional expressions, such as facial expressions or speech, to recognize emotions. However, sometimes the emotional states remain internal and cannot be detected by external expression. In the research reported in this paper, EEG signals were used to identify emotions. EEG is one of the most important techniques used in collecting human brain signals, due to the availability of devices and their high time accuracy. The EEG technology collects the signal directly from the brain using metal electrodes placed directly on the head. Human emotions can be studied through external expressions, such as facial expressions, speech, and body language [12]. Emotions can also be studied through monitoring internal physiological signals that interact and change with the emotional state of humans, through various techniques, such as EEG signals and MEGs [13]. The internal physiological signals are characterized by the fact that they are not affected by self-will. The person cannot control the amount or intensity of these signals during the period of emotion, which give a more accurate estimate of the emotional state. Several studies have shown the superiority of deep learning methods in multichannel EEG-based emotion classification. This study improves the performance-recognition model of emotions using deep-learning algorithms

The brain is the central part of the human body and controls all its organs. The human brain contains the nervous system that provides electrical signals to the human body’s other organs. The primary data processing units in the brain are known as neurons [14]. 

The electrical signals of the brain are processed among the neurons. The EEG signal is acquired by the electrodes and represents brain waves. Multiple channels are used to obtain EEG data with various electrodes [14]. During the emotion recognition process, brain signals are recorded by the electroencephalography (EEG) devices. Deep learning approaches have been used in analyzing the EEG signal. Enhancing the performance and accuracy of emotion recognition from the EEG signals is the key focus of this work, using deep learning approaches [12].

Accuracy is important in the emotion recognition process, especially in analyzing behavioral and psychological disorders, because it helps in making medical decisions. However, it has not been found easy to analyze and classify human emotions, and researchers have observed differences in the accuracy ratios in many studies conducted to identify emotions from EEG signals. Their results differed due to the diversity in many aspects of the research methods, such as variations in experience, the environment, data pre-processing techniques, and classifiers. Hence, it is agreed that there is a need for developing better methods to achieve high performance [15].

To improve the efficiency of the methods used for emotion recognition, researchers must develop novel methods to offer superior performance and reduce complexity. In this paper, an approach for emotion recognition using EEG signals is proposed, which will help doctors in diagnosing psychological or behavioral disorders, with accurate results in a short time. In this study, we aim to improve the model’s performance using a Binary Grey Wolf Optimization (BGWO) algorithm in the feature selection stage to solve the data complexity problem in EEG signals. In addition, this study aims to use a stacked Bi-LSTM classification model to obtain high accuracy in emotion prediction by analyzing EEG signals. The proposed approach includes several phases, which are: data selection, feature extraction, feature selection and classification. 

This paper provides several contributions in the field of emotion recognition. The research contributions can be summarized in the following points:

- A deep learning model was developed by building a network using bi-LSTM to classify multichannel EEG features.
- The model can classify the patterns of multichannel EEG signals that have time and waveform frequency variation; the extracted time-domain-based features and the correlation information clearly improve the model’s performance.
- The Hurst exponent has been adopted as an important feature of EEG classification.
- The methods used in the feature extraction phase reduced model learning and generalization time and reduced the likelihood of overfitting.
- The feature selection phase enhanced the accuracy of the proposed model; as the BGWO algorithm was used, the algorithm contributed to reducing the high dimensions of the dataset and reducing the complexity, which led to a reduction in classification time and an increase in the effectiveness of the model performance.
- This model can perform the classification process of brain signals with high performance and accuracy for biomedical studies. Therefore, its results can be leveraged as a deep learning-based decision support system for medical purposes.

### Related Works

In the study by George et al. [16], the SVM method was also used, with a more accurate overall result of 92%. The DCT method and a box-and-whisker chart were used to determine the features. In the DEAP dataset, containing 32 participants, the researchers concluded that the Fast Fourier Transform (FFT) statistical features for detecting emotions resulted in 92% higher accuracy. Therefore, this method is superior to the technique used in Seeja et al., in terms of the results’ accuracy. The difference between the results is due to the different techniques used in extracting the features and pre-processing the data.

Alhagry et al. [17] discussed the importance of emotion recognition systems that rely on Human–Computer Interaction (HCI) systems. They identified three main problems: the arousal, the valence, and the liking ratio, unlike most studies in this field which discuss only two levels (arousal and valence). Using the DEAP dataset, they extracted features using LSTM- RNN for classification, achieving good accuracy of 85.65%, 85.45%, and 87.99% with the valence, arousal, and liking categories, respectively. It should be noted that they used the end-to-end methods without using feature extraction methods, because deep learning algorithms have the ability to extract features and classify them in the same step.

In another study [18], graph convolutional neural networks (GCNN) were used to implement an emotion recognition model using EEG. The experiment was applied to the DEAP database. After segmenting the data and extracting the differential entropy features, a method known as ECLGCNN, based on the merging of GCNN and LSTM was used. The researchers confirmed the effectiveness of the methods used, as they achieved an accuracy of 90.45% for valence label and 90.60% for arousal in subject-dependent and 85.04% in the independent trials. The complexity of computing required in this method needs to be reduced by developing methods for extracting more features.

The authors in [19] used the end-to-end method to classify emotions using the CNN model, which has demonstrated the ability of efficient feature extraction. This study added additional layers to the CNN model to increase the depth and improve classification capacity. Three datasets, DEAP, LUMED, and SEED, were used in this study. The model achieved 86.56% and 78.3% accuracy in the SEED dataset, 72.81% in the DEAP dataset, and 81.8% in the LUMED dataset.

An emotion recognition model was developed by [20] to identify three emotions (positive, neutral, and negative). Simple recurrent unit (SRU) models were generated using four features across five frequency bands using a SEED dataset. SRU was proposed for several reasons. It can process sequence data and solve the problem of long-term dependencies in RNN. The time, frequency, and nonlinear features were extracted using the dual-tree compound wave transfer (DT-CWT), achieving an accuracy of 80.02%. This model relies on a trial-and-error methodology.

With rapid advances in the emotion recognition field, Chao et al. [21] discussed the problem of multiple channels of electroencephalogram (EEG) signals. They presented an advanced approach to address this problem and proposed a deep belief-conditional random field (DBN-CRF) to develop deep belief networks with glia chains (DBN-GC). The model was applied using three different datasets (AMIGOS, SEED, and DEAP). These methods performed well, with an average accuracy of 76.13%.

Seeja et al. [22] studied the emotional responses to stimuli from EEG signals, using a DEAP dataset and choosing two methods of feature extraction: the Variational Mode Decomposition (VMD) and the Empirical Mode Decomposition (EMD). The researchers also used the DNN method for classifying emotions. This was found to be an effective method, with a valence accuracy of 62% and arousal accuracy of 63%. The study found that the emotional recognition model achieved a better performance with the deep neural network classifier compared to that with the SVM classifiers. The researchers argued that the VMD-based features method offered better performance compared to the EMD-based method and reduces signal complexity. However, the accuracy still needs improvement by improving the frequency resolution of EMD, using various masking operations for the amplitude rate between the mono-components.

Natraj et al. [23] used two types of datasets (DEAP and SEED-IV) and proposed the DWT method to extract the statistical features, frequency domain, the Hurst exponential, and the reciprocal entropy of the signals. The SVM method was used for signal classification. The researchers achieved a valence accuracy of 79% for the DEAP dataset and 76% for the SEED-IV dataset, concluding that the SVM classifier’s channel-merging method yields better results for the DEAP dataset, compared to the SEED-IV dataset. 

Amiri et al. [24] conducted a study to classify emotions in real time, according to the arousal/valence dimensions model, applying the DEAP dataset. The researchers suggested extracting the features of the EEG signals using the DWT method. In this study, there were two different types of classifiers to yield high accuracy: SVM and KNN. This study found that the high-frequency (gamma) band produces higher accuracy than the low frequencies of the EEG signal. The results obtained were comparable, with valence accuracy of 84% and arousal accuracy of 86%.

Numao et al. [25] used the PSD method for feature extraction with the MLP classifier. The researchers were also interested in developing emotion detection using EEG data and used the DEAP database, but focused on the participants’ interaction with music to study emotional responses. The researchers concluded that music affected brain waves at different levels. When the music is unfamiliar to a person, it enhances EEG-based emotion recognition methods. The results achieved 64% valence accuracy and 73% arousal accuracy. This study has a good implementation time, due to the use of MLP (which is a class of ANN), which is suitable for classification prediction problems.

In [26], the researchers discussed the problem of insufficient applications of neural patterns in subjective emotion recognition systems. Researchers collected the signals from 30 participants while they watched 18 videos. When collecting the signals, the researchers concluded that the high-frequency features of EEG signals showed better results using electrodes distributed on the temporal, frontal, and occipital lobes. The researchers classified six main emotions (fear, joy, sadness, disgust, neutrality and anger). The STFT algorithm was used to extract the features, and the SVM method for classification. The study achieved a valence accuracy of 87.36% in discriminating emotions and 54.52% for arousal. Further, in the study of [27] that used the same STFT algorithm for feature extraction with the DEAP dataset, but with the CNN classifier, 83.88% were found with comparable accuracy. Comparing these two studies, it was concluded that the SVM classifier results were more accurate than those of the CNN classifier. These studies still need to add a pre-processing phase to improve the performance.

Girardi et al. [28] studied emotion recognition through biometrics, for use in the health field. The researchers used EEG, EMG, and GSR sensors to collect different types of signals and used them to develop a low-cost emotion recognition model. The study aimed to find the level of valence and arousal in emotions. Using a DEAP database, the study adopted PSD and CSP methods to extract the features and SVM classifier. This study achieved a valence of 56% and arousal of 60%, providing a good solution for the problem of expensive sensors, through low-cost tools. However, the method needs to be developed using the pre-processing of signals to give more accurate results, especially in the medical field.

In another study [29], the accuracy of the Convolutional Neural Network (CNN) results was also verified, as researchers used this to detect the emotional state of humans by analyzing 32 EEG signals. The researchers obtained results with an accuracy of 95.96% for valence and 96.09% for arousal.

In this paper, the performance and efficiency of the emotion recognition model were improved. The proposed approach includes four phases: data selection, feature extraction, feature selection, and classification. The remainder of this paper is divided into three further sections. The Section 2 describes the methods used in this research. The Section 3 presents the experimental results and a discussion of the findings, followed by a summary of the conclusions and future work in the final part.

## 2. Materials and Methods

**Figure 1:**
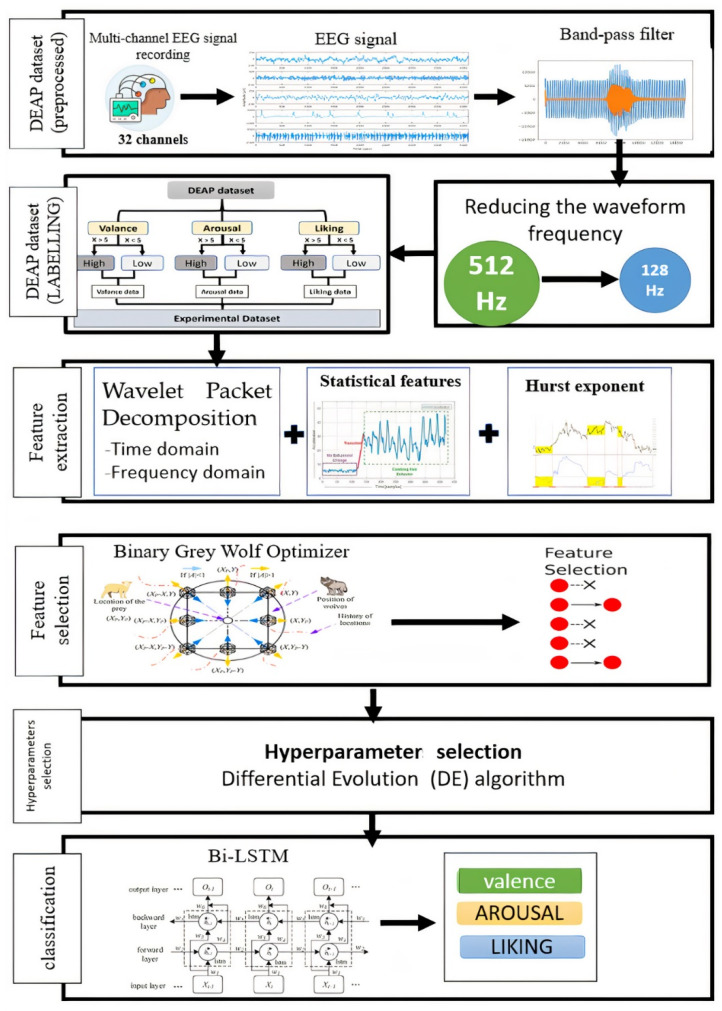
The proposed approach.

In this study, EEG signals were the inputs for the developed model, with emotion detection performed using the deep learning classifier. Key features, such as statistical features, Wavelet features, and Hurst Exponent were extracted from the input signals. The feature selection task was performed using an approach called Binary Grey Wolf Optimizer (BGWO). The selected features were learned by the stacked-layer bi-directional long short-term memory model, which provided the classification of different emotions from an EEG signal. This model was implemented using the MATLAB 2020b software. Figure 1 shows the steps of the emotion recognition model for this paper.

The following sequential techniques were followed to implement the model:

- The DEAP dataset, one of the most popular datasets used to classify multichannel brain signals, was obtained. The preprocessing process was carried out using a high-pass filter to remove noise and filter brain signals. The preprocessing process resulted in a frequency reduction from 512 to 128 Hz.
- The emotion recognition task was divided into three binary classification problems, as follows. The proposed model classifies multichannel EEG into three leading indicators: arousal, valence, and liking. Each division was divided from 1 to 9. Each indicator was divided into two categories. If the rating is less than 5, the rating is set as “low.” If the rating is greater than or equal to 5, the rating is set as ‘high’. Thus, we have six designations: HA (high excitation), LA (low valence), HV (high valence), LV (low valence), HD (high liking), and LD (low liking), in three dimensions.
- The features were extracted from the signals. This step contributed to increasing the accuracy of the classifiers by obtaining the most valuable features from the signals. Several methods were used to extract features, which were the time-domain features, frequency-domain features of signals, Wavelet packet decomposition (WPD), and Hurst exponents.
- The feature extraction phase reduced model learning and generalization time and reduced the likelihood of overfitting.
- After that, the BGWO algorithm was applied in the feature selection stage. It proved its effectiveness in reducing the high dimensions of the data and their complexity, thus giving more accurate results and better performance.
- The hyperparameter was selected using one of the random search algorithms, DE. This step contributes to increasing the accuracy of the classifier by finding the optimal values for hyperparameters.
- In the classification stage, the data were divided into two main groups for training and testing, with a percentage of 70% and 30%, respectively. The Bi-LSTM classifier was used for several reasons:
  ○ It addressed the vanishing gradient problem found in traditional RNN.
  ○ Large sequential data from EEG signals require classification commensurate with the sequential structure of time.
  ○ BiLSTMs have the feature of additional training by training the data in two different directions (from left to right and from right to left), which increases the performance and accuracy of the model.
  ○ BiLSTM works effectively to solve sequence prediction issues and time series forecasting problems.

### 2.1. Data Selection

The DEAP dataset, used by most of the researchers in emotion detection fields, was utilized to validate the model. The dataset was provided by the researchers of the Queen Mary University of London. The number of participants in this dataset was 32. Samples were recorded while watching a music video. Among them, 16 male and 16 female subjects watched 40 music videos selected in terms of different levels of arousal, valence, liking/disliking, dominance and familiarity [25,27,30]. 

The valence and arousal were measured from 1 to 9 (these values are represented by the Circumplex Model. During the signal recording phase, this dataset featured a high EEG recording frequency of 512 Hz. Table 1 contains a summary of the most important information in the dataset [30].

For this paper, the DEAP pre-processed dataset files were used. The files of all the participants contain two matrices—data and label (as illustrated in Table 1). It can be seen in Table 1 that the dataset is divided into two basic matrices in the numerical data and labels. The data matrix has dimensions (40 × 40 × 8064) that contain the channels and video data. The label set has a 40 × 4 dimension of experiences (Valence, Arousal, Dominance, and Liking) [16]. This dataset was pre-processed with a bandpass filter in the (4–45) Hz frequency range. Signals were reduced to 128 Hz, and the noise of the signals was reduced [30].

### 2.2. Feature Extraction Methods

Feature extraction is an important phase in Brain–Computer Interface applications. In this paper, a set of pre-processed data that does not contain noise was used. Feature extraction is helpful in understanding data and reduces the amount of data calculation and storage requirements and the training time. The features were extracted for two reasons: first, it is still possible to extract more useful information than the signal to contribute to accurate results [23] and second, to reduce the data dimensions to better prepare the data for classification and thus increase the classification accuracy in the BCI system. In addition, deep learning models were applied [31].

### 2.3. Hurst Exponent

In the feature extraction process, the Hurst exponent is used to measure long-term memory changes for time series [32,33] and has been used in several fields, including hydrology and genetics. It measures the presence or absence of long-term trends in one-dimensional sequential signals such as EEG signal sequences [34,35]. The following equation measures it:

(1) $$E\left[\frac{R\left(n\right)}{S\left(n\right)}\right]=Cn^{H}\;as\;n\to \infty$$

where:

- [*R*(*n*)/*S*(*n*)] is the rescaled range.
- *E*[*x*] refers to the expected value.
- *n* is the time of the last observation of the input time series data.
- *h* refers to a constant value.

### 2.4. Wavelet Packet Decomposition (WPD)

Wavelet transform allows precise identification of signal components by extracting transducer parameters and functions in the signal. In this study, WPD was used to analyze the recorded EEG signal into multiple resolutions with subsets of parameters [36]. WPD is an alternative method for measuring energy density spectral density. It is used to measure the importance of frequencies in EEG signals and has the advantage of preserving the signal’s time-domain information, which is lost in the power spectral density method [37]. 

In this paper, Wavelet Packet Decomposition was used for wavelet beam analysis. In WPC, the signal parameters are divided to create a binary tree. This type of wavelet transformation has the advantage of being able to pass the discrete-time signal through more filters than the traditional Discrete wavelet transform (DWT).

**Figure 2:**
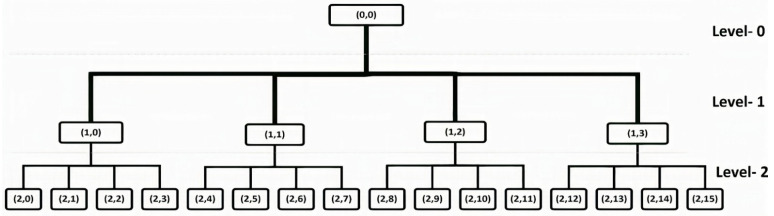
Wavelet Packet Decomposition Tree.

The low-pass and high-pass filters were used to create the binary tree. Reduced signals from HP and LP and these two methods have the advantage of not losing any information [31]. The wavelet method divides the signal frequencies to obtain a tree, as shown in Figure 2. Equations (2) and (3) represent low-pass and high-pass filter coefficients [38,39].

$$\left(W_{n}\left(x\right),\;n=0,1,2,\;\ldots \right)$$

By:

(2) $$W_{2n}\left(x\right)=\sqrt{2}\;\sum_{k=0}^{2N-1}h\left(k\right)W_{n}\left(2x-k\right)$$

(3) $$W_{2n+1}\left(x\right)=\sqrt{2}\;\sum_{k=0}^{2n-1}g\left(k\right)W_{n}\left(2x-k\right)$$

where:

- *W*_0_ (*x*) = *φ*(*x*) represents the scaling function.
- *W*_1_ (*x*) = *ψ*(*x*) represents the wavelet function.

### 2.5. Statistical Features

The EEG signal is a non-stable signal in the time domain. Thus, time-domain features such as statistical data are analyzed, to explain the signal’s properties correctly. A set of statistical features were computed from the EEG signal, such as mean, variance, standard deviation, a deviation that computes the percentage of signal asymmetry around its mean, and kurtosis, which computes the amount of signal tail. The following equations explain the statistical features extracted in this study:

Mean:

(4) $$\mu =\frac{1}{n}\sum_{i=1}{}^{n}x_{i}\;$$

Variance:

(5) $$V=\frac{1}{n+1}\;\Sigma_{i=1}^{n}{\left|x_{i}-\mu \right|}^{2}\;\;$$

Standard Deviation:

(6) $$\sigma =\sqrt{V}=\sqrt{\frac{1}{n+1}\;\sum_{i=1}^{n}{\left|x_{i}-\mu \right|}^{2}}\;$$

Skewness:

(7) $$S=\frac{\frac{1}{n}\;\sum_{i=1}^{n}{\left(x_{i}-\mu \right)}^{3}\;}{{\left(\sqrt{\frac{1}{n}\;\sum_{i=1}^{n}{\left(x_{i}-\mu \right)}^{2}\;}\right)}^{3}}\;\;$$

Kurtosis:

(8) $$K=\frac{\frac{1}{n}\;\sum_{i=1}^{n}{\left(x_{i}-\mu \right)}^{4}\;}{{\left(\sqrt{\frac{1}{n}}\;\sum_{i=1}^{n}{\left(x_{i}-\mu \right)}^{2}\right)}^{2}}\;$$

### 2.6. Feature Selection Method

**Figure 3:**
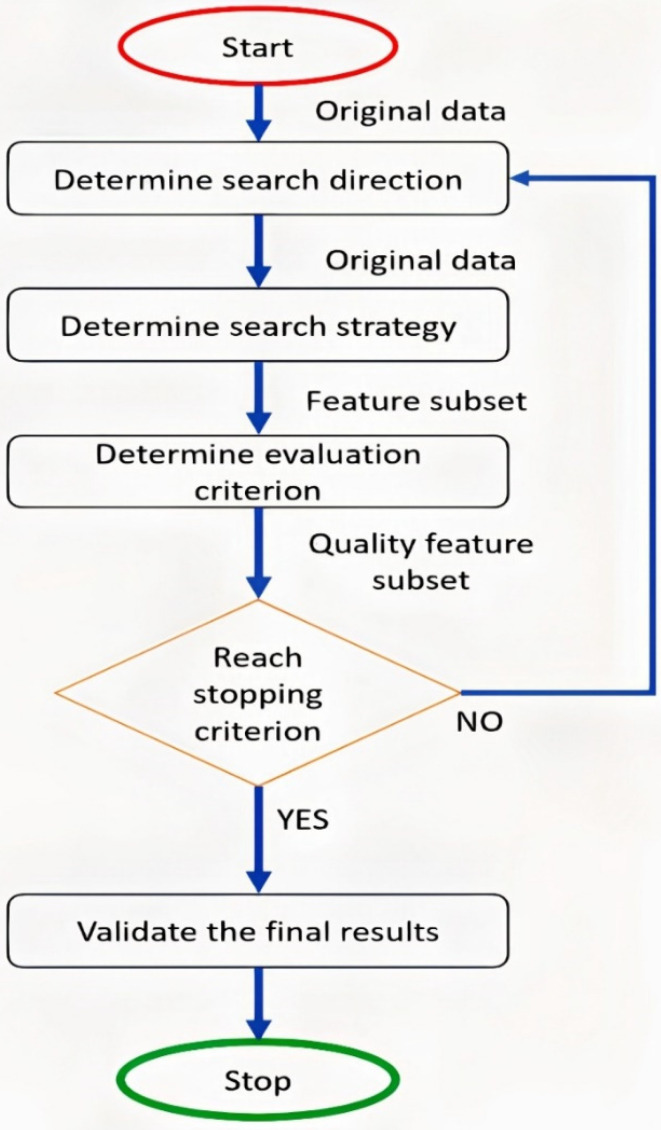
The basic steps of the feature selection process.

In this study, an optimizer to update various parameters to reduce loss [40] was used. The primary goal of the feature selection phase is to reduce the number of input variables in developing the proposed classification model. The optimizer’s primary function is to form and formulate the model in its most proper form by manipulating weights [41]. An optimization algorithm (BGWO) was used to select the features. These details will be indicated in the following sub-sections. This step was used to remove the EEG signal’s redundant features and define a subset’s parameters derived from the base group. The method is characterized by selecting the features without losing information regarding their importance [42]. The filter-based algorithm BGWO relies on defining a subset of features based on its usefulness, and classes are divided into groups Alpha, Beta, Delta, Gamma. The feature selection process is divided into five main stages (as shown in Figure 3).

### 2.7. Binary Grey Wolf Optimization (BGWO)

The results of the study in [43] demonstrate that the GWO algorithm provides competitive results in improving the accuracy of classification. GWO works on high probability and algorithms help generate the optimum solution. In this algorithm, the data is divided into groups alpha (*α*), beta (*β*), delta (*δ*), and omega. Wolves are assigned to these groups, the first three wolves are fittest, *α*, which direct the other wolves (*ω*). During the improvement process, wolves update their positions around *α*, *β*, or *δ* as follows:

(9) $$\vec{D}=\left|\vec{C}\;\cdot \;\vec{X_{p}}\;\left(t\right)-\vec{X}\;\left(t\right)\right|\;$$

(10) $$\vec{X}\;\left(t+1\right)=\vec{X_{p}}\;\left(t\right)-\vec{A}\cdot \vec{D}$$

**Figure 4:**
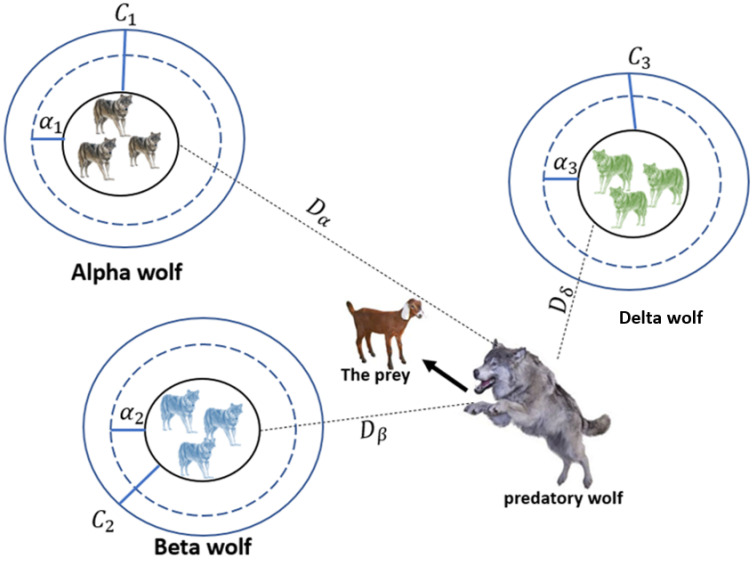
BGWO architecture [43].

Through Equations (9) and (10), wolves can update their position as shown in Figure 4, according to the (*X*, *Y*) coordinates of the continuous space around the valence.

The BGWO assumes that *α*, *β*, and *δ* are the (optimal) prey positions. During the optimization process, the three best solutions assumed to be *α*, *β* and *δ* can be obtained. The following equations can be used to update the position of the wolves, while Equations (11)–(14) are obtained by determining the final position of the wolves [43].

(11) $$\vec{D_{a}}=\left|\vec{C_{1}}\cdot \vec{X_{a}}-\vec{X}\right|$$

(12) $$\vec{D_{\beta }}=\left|\vec{C_{2}}\cdot \vec{X_{\beta }}-\vec{X}\right|\;$$

(13) $$\vec{D_{\delta }}=\left|\vec{C_{3}}\cdot \vec{X_{\delta }}-\vec{X}\right|\;$$

(14) $$\vec{X_{1}}=\vec{X_{\alpha }}-\vec{A_{1}}\cdot \left(\vec{D_{\alpha }}\right)\;$$

(15) $$\vec{X_{2}}=\vec{X_{\beta }}-\vec{A_{2}}\cdot \left(\vec{D_{\beta }}\right)\;$$

(16) $$\vec{X_{3}}=\vec{X_{\delta }}-\vec{A_{3}}\cdot \left(\vec{D_{\delta }}\right)$$

(17) $$\vec{X}\;\left(t+1\right)=\frac{\vec{X_{1}}+\vec{X_{2}}+\vec{X_{3}}}{3}$$

Binary Gray Wolf Optimizer (BGWO) extends the GWO algorithm’s application. It is applied to binary optimization issues. The GWO improved the optimization probability by guiding the wolf position during hunting. The entire food searching phenomenon of the grey wolf is considered a cell space. The cell structure of the grey wolf’s food-searching phenomenon is analyzed based on the cells’ interaction. The cell space is cut by infinite virtual grids, which cannot be subdivided in the future. Each grid provides a single solution [44,45]. The wolf and potential candidate solutions are differentiated by the smart cells. The smart cell is a search space, which represents a wolf’s search space in GWO. When the solution of a cell is not represented, any solution of the best wolf-searching behavior does not come in the category of smart. The smart cells can construct their neighborhood smartly by employing the neighborhood function [46]. The neighbor forces out the wolf from the inner trajectories of the cell. The search space is considered to be a plane, cut by the range of two variables. All the best positions of the wolf (alpha, beta, and gamma) are distributed into the plane and a set of virtual grids constructed to cut the search space. Each grid now contains one of the best solutions to the position of the wolves [47,48].

**Figure 5:**
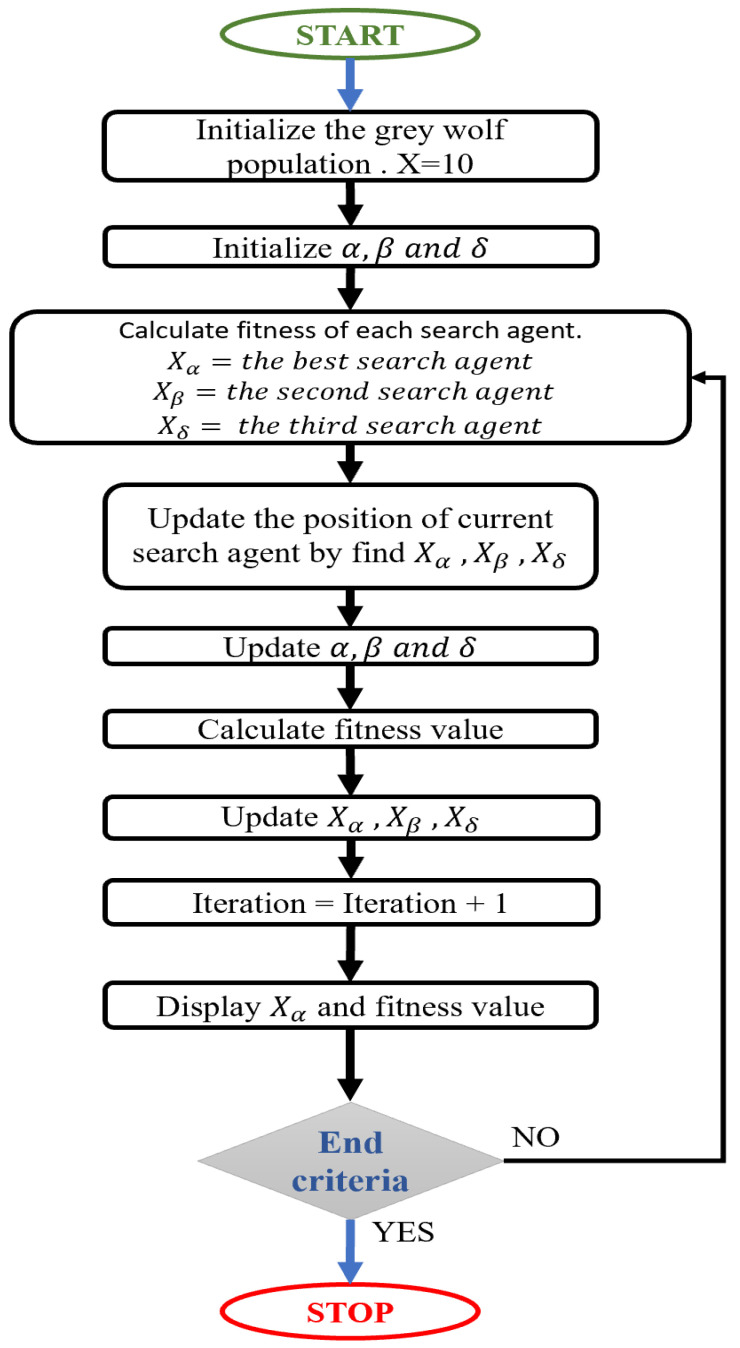
Implementation steps of binary gray wolf optimization.

Recently, numerous meta-heuristic calculations have been created and applied to tackle various estimation issues. The central rationale is that we can tackle the majority of real-life issues by utilizing a legitimate numerical display and calculations to acknowledge them. GWO does not have many boundaries to tune and has a decent harmony between investigations; thus, GWO is basic, simple to utilize, adaptable, and versatile. Figure 5 shows the BGWO Algorithm flowchart.

### 2.8. Classification Method

The classification belongs to the category of supervised learning. In the classification stage, a prediction is made for a specific data category called classes or labels. In this phase, the Stacked Layer Bi-LSTM (Bi-Directional Long Short-term Memory) model was trained with the optimal selected features and classified the emotions. The use of deep learning models used for signal classification purposes has increased dramatically. The LSTM algorithm has shown its effectiveness in automatically predicting timeline properties and remembering important information or values for a longer time. Typically, a stacked Bi-LSTM network is used to process and classify time series and sequence signals accurately [17].

### 2.9. Bi-Directional Long Short-Term Memory

The LSTM classifier consists of four main components: memory cell, input gate, forget gate, and output gate. The memory cell stores data for a long or short time. The Input Gate controls the amount of information, while the Forget Gate is used to control information retention in the LSTM cell. The LSTM layer cell’s information can be controlled to calculate and format the output activation for the Output Gate [49,50].

**Figure 6:**
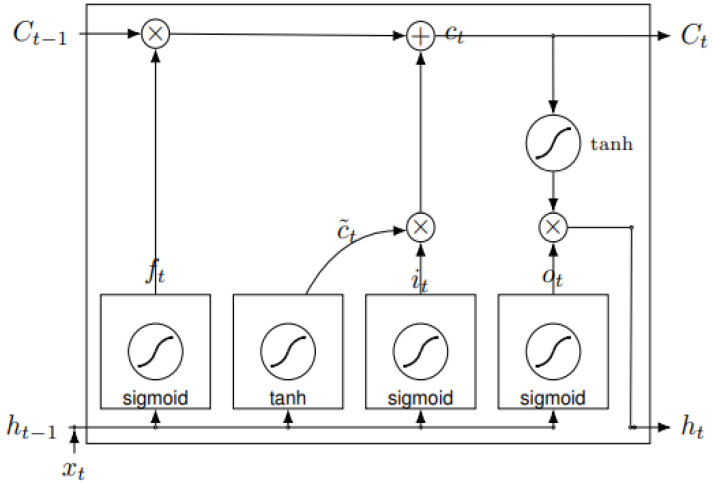
Cell structure of Bi-LSTM. Source: [17].

LSTMs Networks are an extraordinary type of RNN and were presented by Hoch Reiter and Schmid Huber in 1997, to cope with the issue of long-term exploding and vanishing gradients in RNN [51]. Long successions can be hard to obtain from standard RNN, because they are prepared by back-proliferation through time (BPTT), which causes the issue of exploding/vanishing gradients. To resolve this, the RNN cell is supplanted by a gated cell as a Bi-LSTM cell. Figure 6 shows the fundamental engineering of Bi-LSTM cells [31].

The data is entered into a cell case by using three entryways. The first entryway is an overlook door to choose what data to discard from the cell state; a sigmoid layer makes this choice, as in the following equation.

(18) $$f_{t}=\sigma \;\left(W_{f}\cdot \;\left[h_{t-1},x_{t}\right]+b_{f}\right)\;$$

The subsequent door is an input entry way that comprises a sigmoid layer to choose the values to be refreshed, and the tanh layer makes a vector of new refreshed values as depicted in the following equations.

(19) $$i_{t}=\sigma \;\left(W_{i}\cdot \;\left[h_{t-1},\;x_{t}\right]+b_{i}\;\right)\;$$

(20) $$\underline{C_{t}}=tanh\;\left(W_{c}\cdot \left[h_{t-1},x_{t}\right]+b_{c}\right)\;\;$$

The cell state is then updated from Equations (18)–(20), thus.

(21) $$C_{t}=f_{t}\cdot C_{t-1}+i_{t}\cdot {\underline{C}}_{t}\;$$

Finally, the output of the present state will be determined based on the refreshed cell state and a sigmoid layer that chooses the parts of the cell state, which will be the last output represented.

(22) $$o_{t}=\sigma \left(W_{o}\cdot \left[h_{t-1},x_{t}\right]+b_{o}\right)\;$$

(23) $$h_{t}=o_{t}*tanh\left(C_{t}\right)\;$$

where:

- σ  represents the sigmoid activation function.
- tanh represents the tangent activation function.
- W denotes the weight matrices.
- $x_{t}$ is the input vector.
- $h_{t-1}$ denotes the past hidden state.
- $b_{f}$, $b_{i}$, $b_{c}$, $b_{o}$ are biased.

**Figure 7:**
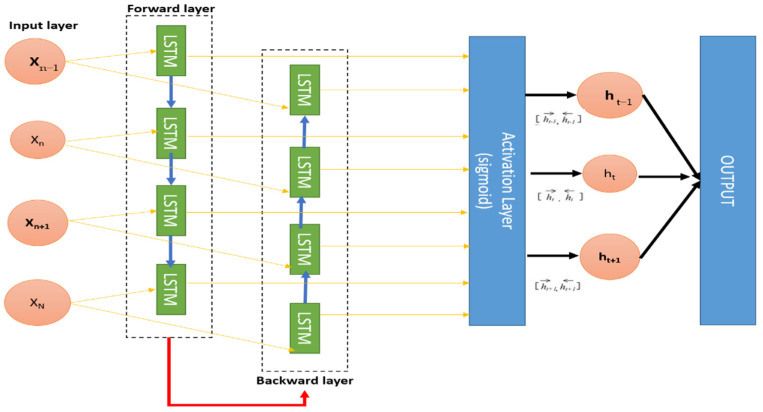
Structure of Bi-LSTM. Source: [52].

In this paper, we use the developed Bi-LSTM. It is a deep learning algorithm that feeds the input sequence into the normal time order of one network and the reverse chronological order of another network. The outputs of the two networks are sequential at each time step, as shown in Figure 7. The stacked layer Bi-LSTM architecture allows both background and forward information about the sequence at each time step to be obtained, thus giving high classification accuracy [52].

Equations (24)–(26) illustrate the way in which the bi-LSTM classifier handles data back to forward.

(24) $$h_{t}{}^{\to }=f\left(w_{1}\;x_{t}+w_{2}\;h_{t-1}{}^{\to }\right)\;$$

(25) $$h_{t}{}^{\leftarrow }=f\;\left(w_{3}\;x_{t}+w_{5}\;h_{t+1}{}^{\leftarrow }\right)\;$$

(26) $$O_{t}=g\left(w_{4\;}h_{t}{}^{\to }+w_{6}\;h_{t}{}^{\leftarrow }\right)\;$$

### 2.10. Evaluation of Proposed Methods

The performance of this model was measured by a set of criteria including accuracy, precision, recall and f-score. Detection errors were measured through the confusion matrix. The performance results of the proposed method were compared with the results of other existing models. 

#### 2.10.1. Accuracy

Accuracy is the most common criterion for evaluating the performance of a classification model. Classification accuracy is calculated according to Equation (27) by computing the ratio of true results to the total number of results.

(27) $$\mathrm{Accuracy}=\frac{\mathrm{TP}+\mathrm{TN}}{\mathrm{TP}+\mathrm{TN}+\mathrm{FP}+\mathrm{FN}}\;$$

#### 2.10.2. Precision

In the classification stage, precision is calculated through Equation (28), which indicates the number of true positives divided by the total number of items belonging to the positive class.

(28) $$\mathrm{Precision}=\frac{\mathrm{TP}}{\mathrm{TP}+\mathrm{FP}}\;$$

#### 2.10.3. Recall

The recall is calculated by computing the sum of the true positives divided by the sum of the true positives and the false negatives, as shown in Equation (29).

(29) $$\mathrm{Recall}=\frac{\mathrm{TP}}{\mathrm{TP}+\mathrm{FN}}\;$$

#### 2.10.4. F-Score

F-score is a method that combines precision and recall. It calculates the harmonic average of the precision and recall model, as shown in Equation (30).

(30) $$F_{1}=\frac{2\;*\;\mathrm{precision}\;*\;\mathrm{recall}}{\mathrm{precision}+\;\mathrm{recall}}\;$$

## 3. Results and Discussion

### 3.1. Feature Extraction from EEG Signals

The feature extraction stage affects the field of the Brain–Computer Interface (BCI), helps implement powerful classification and produces more accurate results. There are two classes of features extracted from the EEG signal, related to the time domain and the frequency domain; the features extracted for EEG signal analysis are shown in Table 2. Wavelet packet decomposition analysis was performed on two major levels. The feature vector was calculated for each channel and stored in a variable to classify the 32 channels used in the next step.

The total number of features extracted from the EEG signals data was 68. In this study, the statistical features of the EEG signal that were extracted included mean, minimum, maximum, skewness, standard deviation, kurtosis, Wavelet Packet Decomposition features and the Hurst exponent. It took 5 h to extract the features from the original dataset.

### 3.2. The Model Training

In this paper, profound learning neural organization is applied to the crude EEG signs of 32 participants who observed the 40 recordings, to perceive the feeling evoked by these recordings. Each video was fragmented into 12 portions, with a length of 5 s. The DEAP dataset was utilized to check the calculation in this work. 

Implementation of Bi-LSTM can learn long-term conditions between the time steps of arrangement information. These conditions can be valuable when the model needs the organization to gain from the total time arrangement at each time step. This model uses the bi-directional LSTM layer (Bi-LSTM) to succeed in both forward and reverse ways [53].

### 3.3. Hyperparameter Tuning Selection

**Figure 8:**
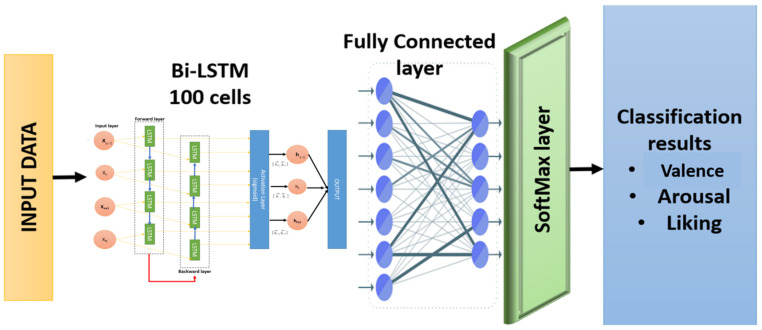
The main training layers.

As shown in Figure 8, the five layers for training the classifier were determined and the ‘Max Epochs’ was set to 35 to permit the model to make 35 ‘goes’ through the training information. A ‘Mini Batch Size’ of 80 guides the organization to attempt 150, preparing signals all at once. An ‘Initial Learn Rate’ of 0.01 assists speed by increasing the preparation cycle. ‘Gradient Threshold’ was set to 1 to balance the preparation cycle by keeping slopes from becoming excessively enormous and ‘Plots’ were determined as ‘training progress’ to produce plots that show realistic preparation progress as the quantity of cycle increments. Table 3 summarizes the classifier settings.

The random search method depends on selecting a random sample of data and using random sets of parameters to reveal the best solutions that provide better performance for the proposed model [54].

One of the most critical challenges in this study is finding an accurate classification method. Parameters play a significant role in the accuracy of the classifier results, such as learning rate and the number of hidden layer units. For example, if a low learning rate value is set, slow convergence will result. Otherwise, the performance will be erratic and unstable. The number of hidden layer units will affect fitting.

The batch size affects the training dynamics. If the batch size is large, it will lead to poor generalization and increase the memory required to perform the training. However, if the batch size is too small, this will lead to convergence in the training data.

In this work, a reliable heuristic random search algorithm is used to determine the optimal values of parameters, which balances the performance and computational efficiency of the BiLSTM classifier. This algorithm is implemented by studying the root mean square error (RMSE), which is the sum of the squared deviations of the expected value $\tilde{y_{i}}$ and the real observed value $y_{i}$ in the regression analysis (representing the focus of the data around the fitting line). RMSE is calculated by Equation (31):

(31) $$\mathrm{RMSE}=\sqrt{\frac{1}{S}\;\sum_{i=1}^{s}{\left(\tilde{y_{i}}-y_{i}\right)}^{2}}\;$$

The Differential Evolution (DE) algorithm follows several sequential steps, starting with the initialization of several factors: the number of iterations, *g*, the size of the population, the crossover rate, and the measure or mutation factor. The population is then generated randomly, as in Equation (32).

**Figure 9:**
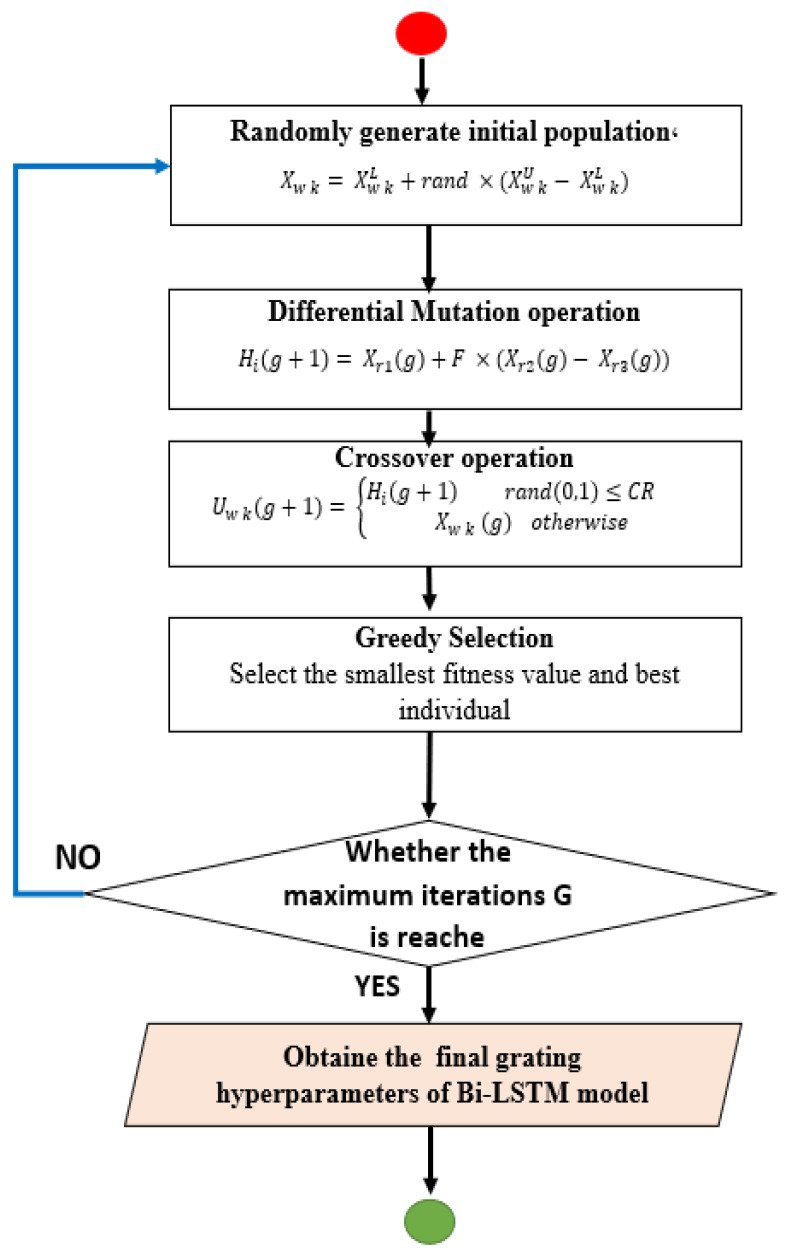
Flowchart of DE algorithm.

The mutation vector $H_{i}$ for the individual population is then generated by Equation (33). Next, we set the intersection process, which is done by selecting individuals randomly, as in Equation (34). Finally, the selection process is performed by Equation (35). Figure 9 shows, in detail, the sequence of operations used in DE to determine the best parameters of the Bi-LSTM classifier.

(32) $$X_{w\;k}=X_{w\;k}^{L}+rand\times \left(X_{w\;k}^{U}-X_{w\;k}^{L}\right)\;$$

(33) $$H_{i}\left(g+1\right)=X_{r1}\left(g\right)+F\times \left(X_{r2}\left(g\right)-X_{r3}\left(g\right)\right)\;$$

(34) $$U_{w\;k}\left(g+1\right)=\{\begin{array}{c} H_{i}\left(g+1\right)\;rand\left(0,1\right)\leq CR\;\\ X_{w\;k}\;\left(g\right)\;otherwise \end{array}\;$$

(35) $$X_{w\;k}\left(g+1\right)=\{\begin{array}{c} U_{w\;k}\left(g+1\right)\;if\;f(U_{w\;k}\left(g+1\right)\;\leq f\left(X_{w\;k}\left(g\right)\right)\\ X_{w\;k}\left(g\right)\; \end{array}\;\;$$

**Figure 10:**
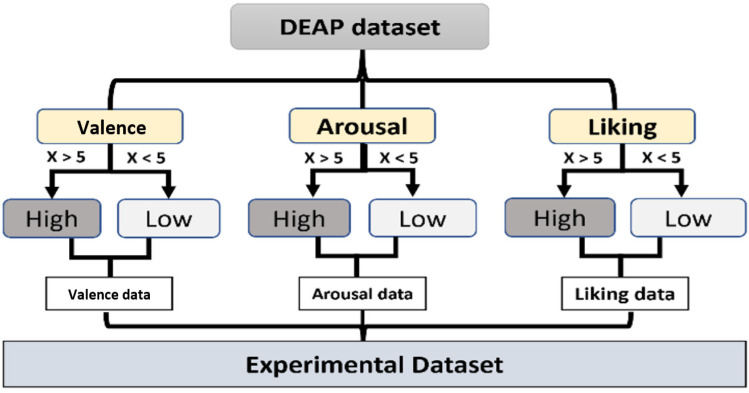
Structures of the categorized dataset.

For Bi-LSTM training, 70% of the total data was used, i.e., an 896 matrix of features (as described above) and the model tested on the remaining data (384). The model was trained for 45 epochs (560 iterations). The network was trained and tested for valence, arousal and liking, as in the raw data, with the value of each emotion from 1–9, and two labels created for each emotion; greater than 5 meant high and less than 5 meant low (as shown in Figure 10), which would make it a binary classification problem. 

To train this model, the ADAM option was used. ADAM is an alternative optimization algorithm for randomized gradient ratios, used to train a deep learning model in less time. In this work, ADAM was chosen because it combines the advantages of the AdaGrad and RMSProp algorithms. ADAM helps to improve the handling of sporadic, random gradients and noise problems. The optimizer provides computational efficiency in training the Bi-LSTM model and is suitable for solving large data or parameter issues [55].

**Figure 11:**
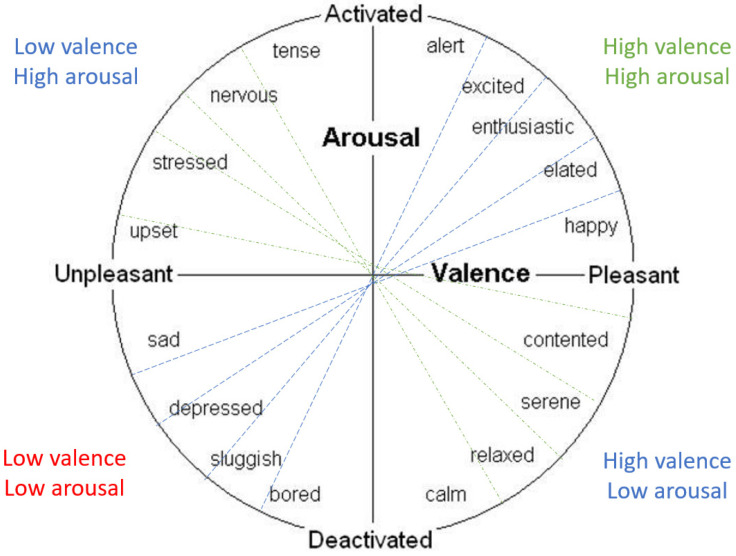
Circumplex Model. Source: [51].

Most of the studies reviewed classify emotions by the percentage of the Circumplex model, because this model is clear and comprehensive. EEG signal classification methods allow the emotion recognition model to produce accurate results. Figure 11 shows the Circumplex model; a person whose signals are raised to low arousal levels and negative valence is more likely to be sad, while a person whose signals are accelerated to higher arousal levels and positive valence is more likely to become irritable or agitated [13].

In this paper, the results are categorized according to the two-dimensional Circumplex model designed by James Russell in 1980, through which it is possible to explain the concept of valence and arousal on which to classify emotions, as shown in Figure 11. This model represents a set of data (emotions) and shows the points of relevance of emotions to one another. It was created to demonstrate that feelings are differentiated, and not completely separate from one another. It is divided into two orthogonal axes, the horizontal axis representing valence, and the vertical axis, arousal. The Circumplex model is ideal for measuring emotional states because it shows them all along with their relative relationships. The horizontal axis of the pattern represents the emotion pattern, such as happiness and sadness. The vertical axis represents the continuity between high and low arousal. In this study, the liking label was also measured (liking means: The amount of a person’s preference for something). It represents the classified response of the participant in the dataset (like or dislike) [56].

### 3.4. Classification for Valence Label 

**Figure 12:**
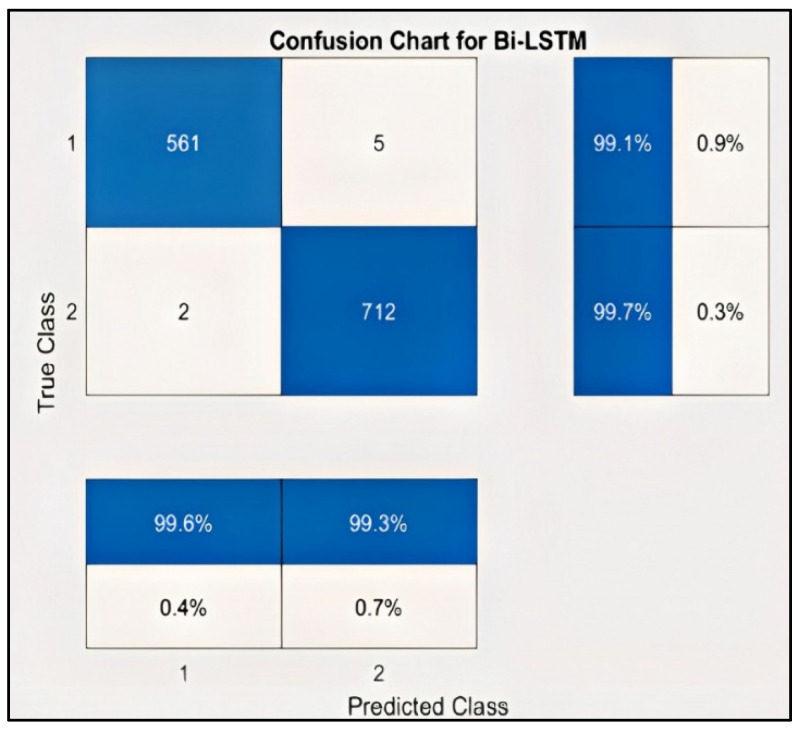
Confusion chart for valence label.

**Figure 13:**
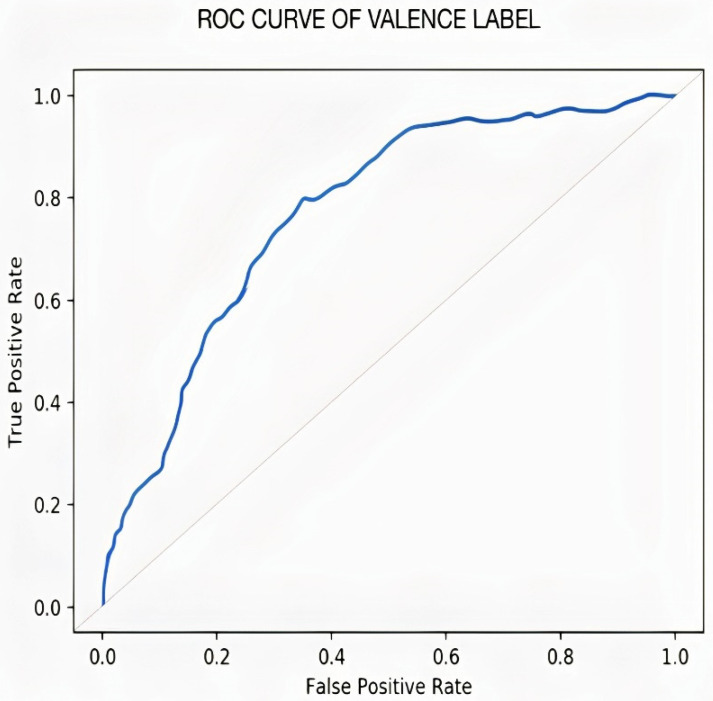
ROC curve of valence label.

**Figure 14:**
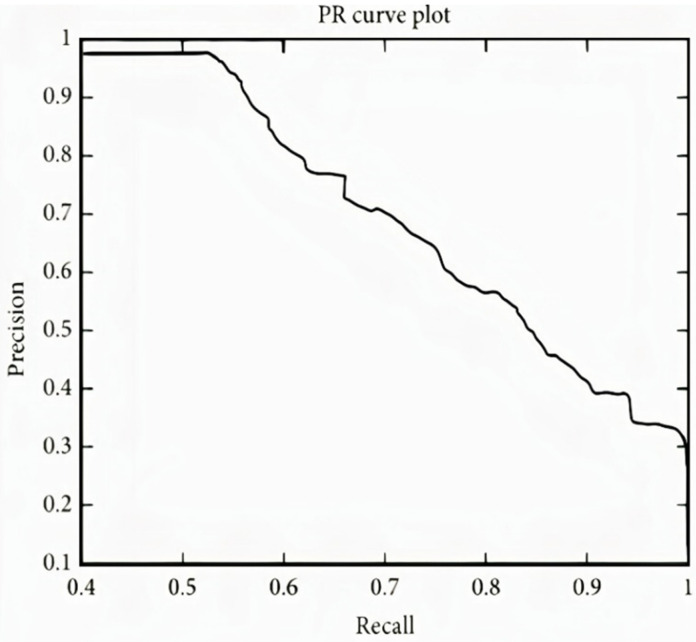
PR curve of valence label.

**Figure 15:**
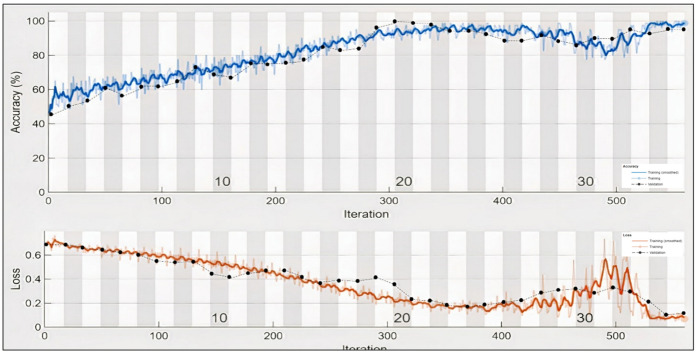
Training progress of Valence label.

Figure 12 represents the result of the stacked Bi-LSTM for the valence label, as it could be observed that the number of misclassifications for the first class is 5, that is 0.9% of first-class data. Similarly, the number for misclassifications for the second class is 2, or 0.3% of second-class data. Here, misclassification means that the network identifies that particular data point as a different class, in place of the original. Overall, the accuracy is 99.6% for the first-class prediction and 99.3% for the second class. This analysis is only for valence emotion. Table 4 shows the performance criteria of the Valence level. Figure 13 and Figure 14 represent the ROC curve and PR curve, respectively. Figure 15 shows the training progress of the model accuracy and the loss rate of the Valence label. 

### 3.5. Classification for Arousal Label

**Figure 16:**
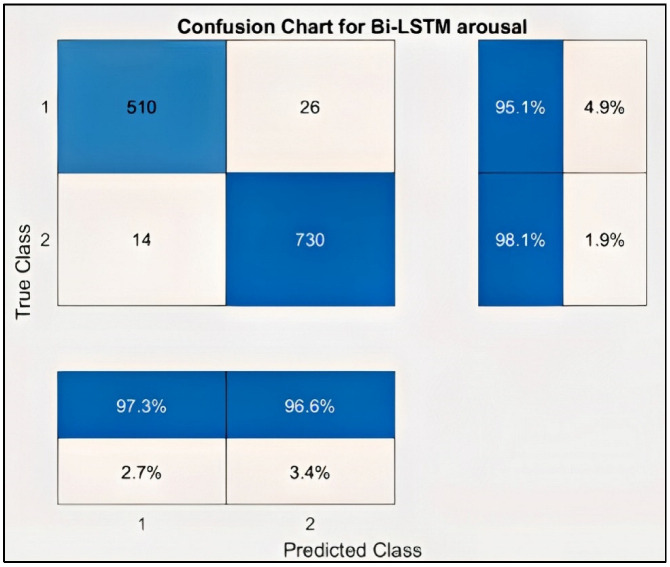
Confusion chart for Arousal label.

Figure 16 represents the confusion chart for the Arousal label and the result of the Bi-LSTM for the arousal emotion, respectively. Similarly, the model was trained for the arousal emotion; as discussed above, the binary label was created for arousal, with less than five indicating low arousal and greater than or equal to five minutes indicating high arousal. Table 5 shows the performance criteria of the arousal level.

**Figure 17:**
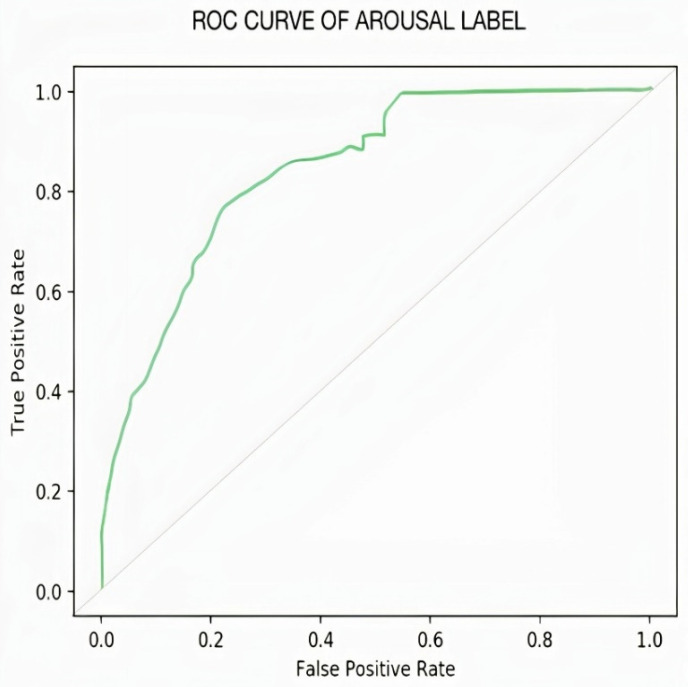
ROC curve of Arousal label.

**Figure 18:**
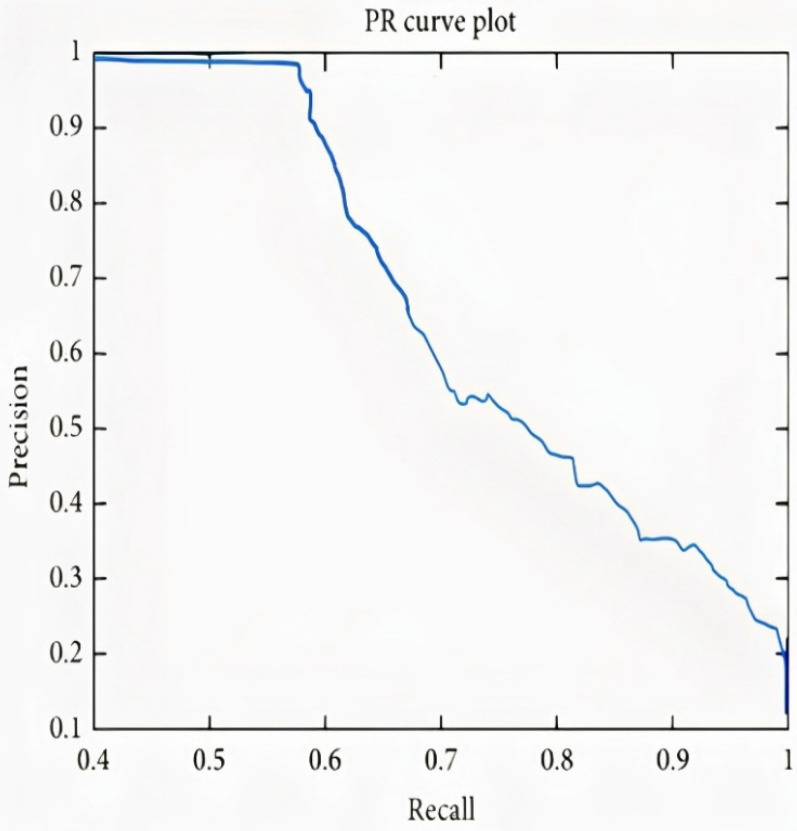
PR curve of Arousal label.

**Figure 19:**
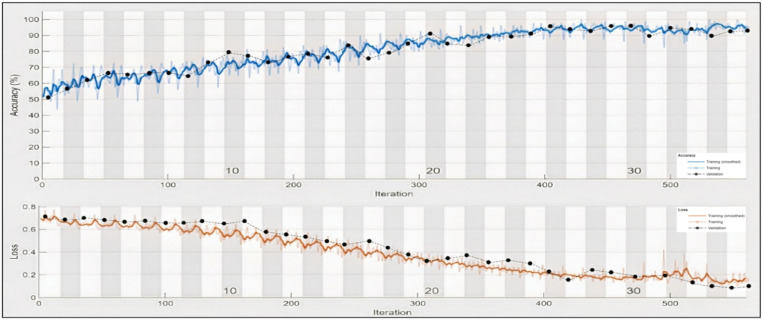
Training progress of Arousal label.

In the case of Arousal-type label data, the accuracy of the Bi-LSTM network is 96.8750%. Through the confusion matrix, it can be seen that the number of misclassifications for the first group is 26 (4.9% of first group of data) and similarly, the number of misclassifications for the second class is 14 (1.9% of second-class data). Here, misclassification means that the network identifies that particular data point as belonging to a different class, in place of the original. Overall, the accuracy is 97.3% for first-class prediction and 96.6% for the second class. Figure 17 and Figure 18 represent the ROC curve and PR curve of the Arousal label, respectively. Figure 19 shows the progress of the model’s accuracy and the loss rate of the Arousal label.

### 3.6. Classification for Liking Label

**Figure 20:**
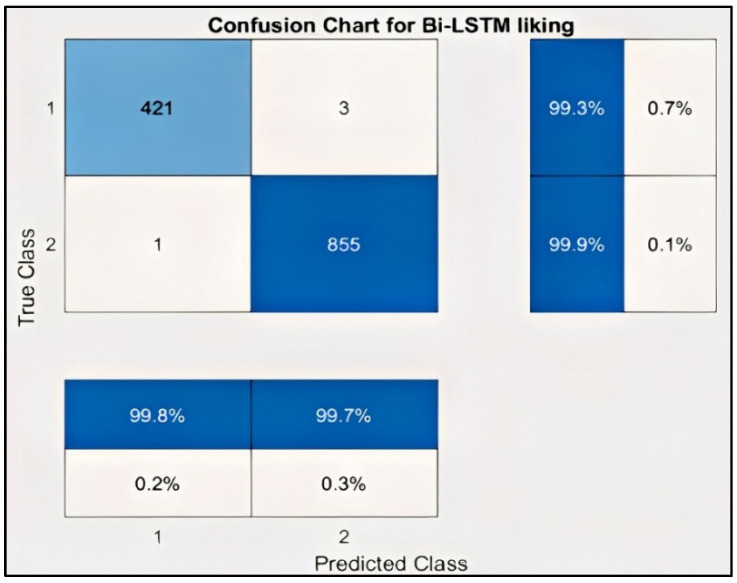
Confusion chart for Liking label.

Figure 20 represents the result of the stacked Bi-LSTM for the liking emotion. Similarly, the model was trained for the liking emotion, obtaining an accuracy of 99.68%. As discussed above, a binary label was created for Arousal, with less than 5 indicating low arousal and greater than or equal to 5 referring to high arousal. Table 6 presents the results for the Liking label.

**Figure 21:**
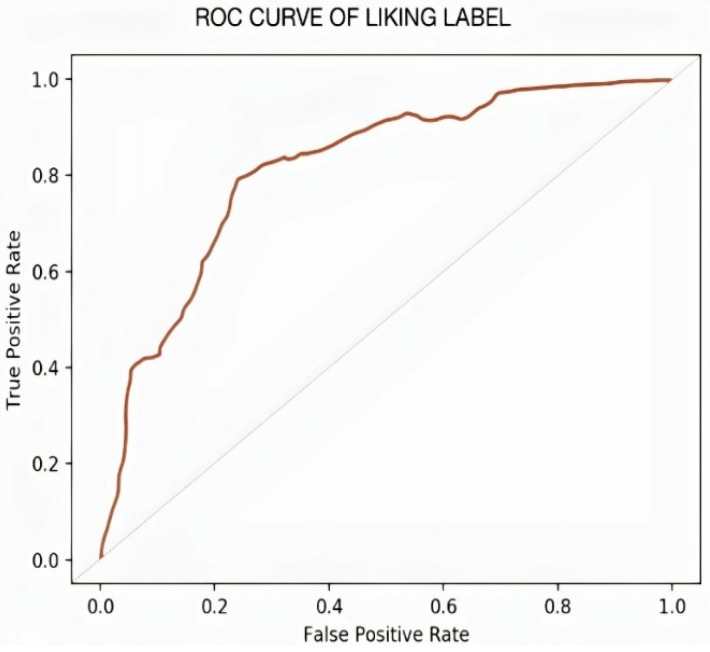
ROC curve of liking label.

**Figure 22:**
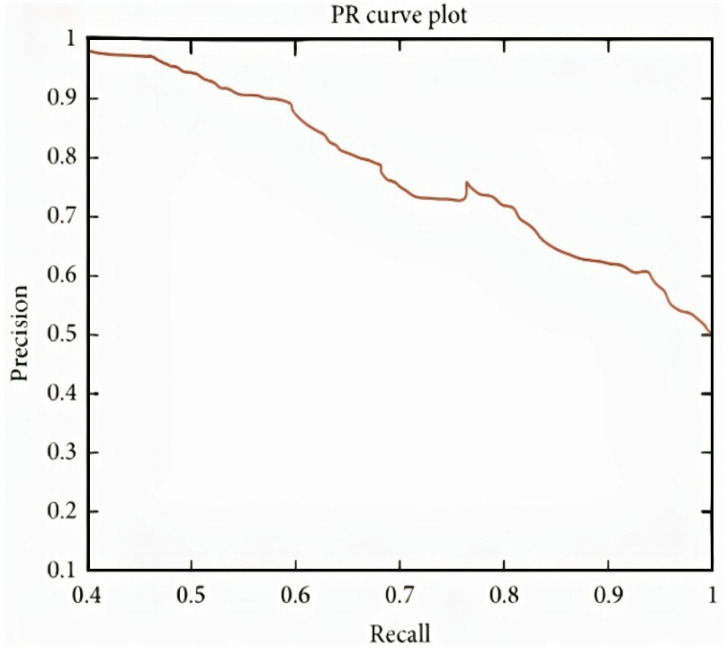
PR curve of liking label.

**Figure 23:**
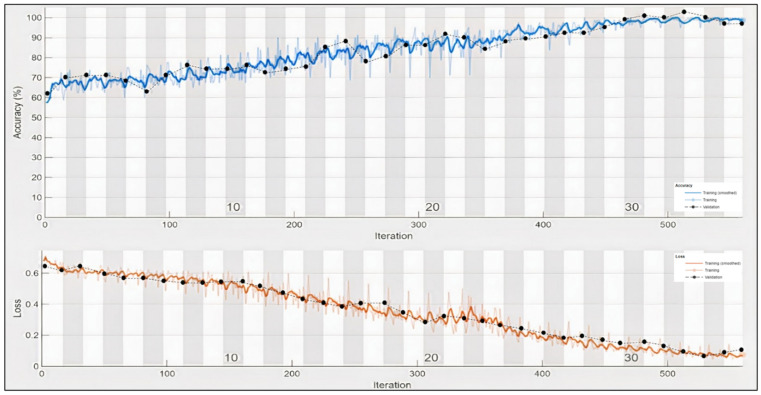
Training progress of liking label.

It can be observed in the confusion matrix that the number of misclassifications for the first class is 3 (1.8% of first-class data); similarly, the number for misclassifications for the second class is 1 (0.1% of second-class data). Here, misclassification means that the network identifies that particular data point as a different class in place of the original. Overall, the accuracy for first-class prediction is 99.8%, and for the second class, 99.7%. Figure 21 and Figure 22 show the ROC curve and PR curve of the arousal label, respectively. Figure 23 shows the progress of the model’s accuracy and the loss rate of the Liking label.

## 4. Discussion

Most BCI systems suffer from a lack of ability to interpret information and emotional intelligence. Accuracy is essential in this area, as it contributes to making a correct decision and appropriate actions. The goal of affective computing is to bridge this gap by precisely classifying emotional responses using emotional cues. This study answered the research question, and the proposed model resulted in high performance in emotion recognition.

In studies of the past, facial expressions or voice were used to elicit emotions. However, these traditional methods do not produce accurate results for the real condition of a person, because the person is able to control their facial expressions and the tone of their voice. In the current study, physiological EEG signals were used, since human beings cannot control them, thus, producing real results for the person’s psychological state. This model was developed using the DEAP dataset for emotion recognition. The model achieved accurate classification effects of 99.45%, 96.67% and 99.68% for Valence, Arousal and Liking, respectively.

In this model, a deep learning method is adopted to process the input. Although deep learning models deal directly with input, the steps were used to choose the feature or reduce the dimensions to increase the performance efficiency of the proposed model. BCI technology depends on several main steps, namely signal collection, pre-processing, feature extraction, and classification. Table 7 presents the results of the statistical tests that prove the significance of the feature selection stage and the effectiveness of the proposed classification model.

When comparing this work with earlier works, this study provides a good analysis of the multi-frequency EEG signal. Attention was paid to the feature extraction and selection stages because they reduce the amount of dimensionality of input data and increase the accuracy of models by removing the redundant data, thus, increasing training speed. Unlike previous studies that extracted only one type of feature, three different types of features were extracted in this study (Hurst exponent, wavelet features, and statistical features). It was concluded that the higher frequency bands, gamma and beta (12–30 Hz), yield more favorable results for the emotion recognition model than other lower frequency bands, such as delta (0–4 Hz), and, thus, high performance was obtained in terms of accuracy, precision, recall, and f-score. 

Many studies in the field of emotion recognition do not include the feature selection stage. However, we believe this to be important for removing duplicate data from the extracted data, reducing data dimensions and data complexity. In this study, the BGWO algorithm was used to select the features. This feature contributed to a significant increase in the efficiency of the model.

In the classification stage, a special type of RNN, the Bi-LSTM, was used. The Bi-LSTM network is good at manipulating the temporal change characteristic of different frequencies in the serial data. In the proposed model, the running time of each label is approximately 37 min. Our model training resulted in high performance and processing efficiency for emotion classification, as shown in Table 8 (summary of the performance criteria results for the proposed model).

**Figure 24:**
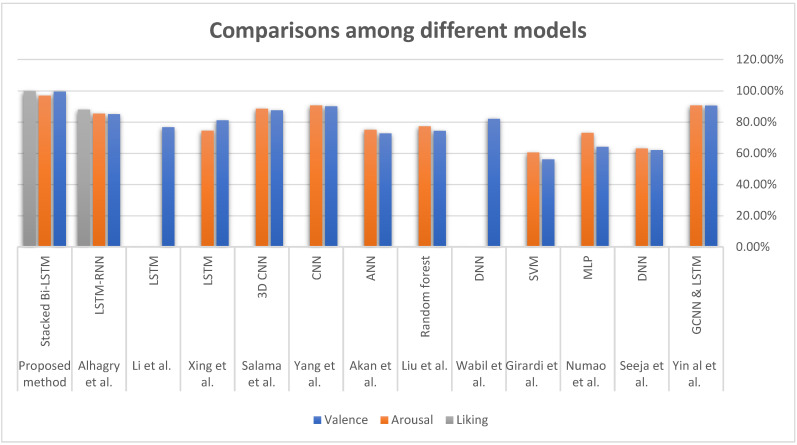
Comparisons among different emotion recognition models [4,17,18,22,25,28,57,58,59,60,61,62].

Recognizing emotions is an essential step in the Human–Computer Interaction process. The results of this study can serve as a reference for researchers working on related applications. Deep learning has proved effective in categorizing feelings, although this differs from machine learning in that it contains more layers and is able to process large amounts of data with high efficiency. When the model relies on learning from sequential data (such as EEG signals), the purpose is to capture the temporal dynamics that allow generalization of time sequences by sharing parameters over time, rather than re-learning them at each step, and this helps the parameters to be shared more deeply. Figure 24 presents a comparison between the different models results for classifying emotions. 

Further, the feature extraction process is vital in BCI applications. Therefore, in this experiment, various feature extraction techniques were selected, such as statistical features, wavelet features, and the Hurst exponent, giving a total number of 68 features.

In the feature selection phase, the binary GWO algorithm was used, which significantly improved the performance of the model. The BGWO has proven its effectiveness in providing competitive results by contributing to the accuracy of rating and approximation of the proposed optimal solution. The BGWO has double exploration and exploitation processes that help the classifier to investigate the efficiency of the algorithm. This algorithm is characterized by its simplicity and speed, as it works by converging towards the optimal solution, and the convergence is very fast.

One of the main reasons for the high classification result in this model was the use of the BGWO algorithm, which has adaptive parameters to effectively balance exploration and exploitation. Half of the iteration is for exploration and the rest for exploitation. The binary GWO algorithm preserves the three best solutions obtained at any stage of optimization and is, hence, able to yield more accurate results due to its high exploration behavior. The highly exploitative behavior of the algorithm is an important reason why a BGWO-based coach is able to rapidly converge towards an optimal level of the dataset. Further, BGWO is recommended when the dataset and the number of features are large due to the large number of local options. A Bi-LSTM algorithm, one of the best deep learning algorithms used to process time series, was used in the classification stage. The Bi-LSTM model outperformed the traditional LSTM used in other studies [57,58]. As the current study showed more accurate results and better effectiveness, the ADAM optimizer was used to increase the efficiency of deep learning algorithm training. ADAM was used to improve the features of the Bi-LSTM algorithm by changing weights and learning rates for the purpose of minimizing losses. Consequently, results were obtained more quickly, with less loss and increased accuracy, as is evident in the training progress model. ADAM maintains the average decay rate of previous gradations, apart from correcting for vanishing learning rate and high contrast. This model has achieved good accuracy of 99.45%, 96.87%, and 99.68% for Valence, Arousal, and Liking, respectively. The deep learning algorithm (Bi-LSTM) achieved better results than the other classifiers used, when compared with the results mentioned in the previous works. 

In Table 8, the accuracy of the results of the proposed model is compared with that of other deep learning and machine learning methods that use the DEAP dataset. The results of the current model showed a significant improvement over the earlier ones, due to the use of an improved approach, vis-a-vis the traditional LSTM used in the studies in [17,57,58].

By comparing the accuracy of the results of previous studies, we conclude that although the dataset is the same, there are different levels of classification accuracy, due to the different techniques of extracting features from EEG signals, the different methods of classifying EEG data, and their different parameters. It is worth noting that the use of the optimizer is of great importance in improving the performance of the model. In most studies, emotions were classified on the basis of Valence and Arousal; in this study, however, Liking was also classified. The research hypothesis can be tested by reviewing the results for the model’s performance criteria (accuracy, precision, recall, and f-score), presented in Table 9, which shows the high performance of the proposed model.

We faced a few challenges while developing the model. The model took a long time for training. The Bi-LSTM algorithm showed sensitivity and complexity in adjusting the O(w) random weight initialization process. These challenges are related to the issues of execution time consumption, reducing the high dimensionality and complexity of the dataset and its naming. In resolving these challenges, we came up with several effective methods for extracting statistical features, wave and time frequencies, and methods for selecting features and creating the correct classifier. We also faced a challenge in determining exact parameter values that would provide a high level of accuracy for the proposed classification model. After several experiments, we came up with a random search method that measures the effectiveness of the proposed parameters of the model.

## 5. Conclusions and Future Work

The task of emotion recognition faces many challenges due to the instability and complexity of EEG signals. This research provided an effective solution for emotion recognition models. The deep learning-based approach was proposed to improve the accuracy of emotion recognition based on EEG signals, using a deep learning algorithm. This study contributed to enhancing accuracy and performance in the field of emotion recognition through the developed algorithms, which had not been used before in this field, such as the BGWO algorithm used in the feature selection phase and the newly developed Bi-LSTM technique. The proposed approach was tested on a DEAP dataset, and classification was implemented with the stacked Bi-LSTM deep learning algorithm. The feature extraction and selection stages improved model performance by reducing data dimension and complexity. Moreover, the method in this study provides a computational model that can quantify the correlations between EEG signals, frequency bands and emotions. The performance of the proposed model was compared with other models that used machine learning, and the proposed model achieved high accuracy in classifying the internal feelings of physiological signals based on the electroencephalogram, using the deep learning model with random search algorithms, which contributed to determining the most accurate parameters and stages of extraction and selection of features of the input signals. Three emotional measures of Valence, Arousal, and Liking were targeted for recognition by the proposed model, which high accuracy of 99.45%, 96.87%, and 99.68% for Valence, Arousal, and Liking, respectively. Comparison of the experimental results of the proposed model with those of the previous studies revealed the former’s superiority in accuracy and performance; further, this model produced competitive results in the field of EEG-based emotion recognition. 

Real state data remain difficult to collect and work on immediately due to the difficulties in creating a dataset, such as the high cost, and limitations of EEG recorders and human resources. In addition, it must be determined whether short videos can provide adequate stimuli to feelings, and whether the emotional volatility of the subjects overlapped during the interval between any two videos. In future research, other classification algorithms can be applied on different datasets to prove their effectiveness in emotion recognition by using advanced deep learning models based on RNN algorithms, such as the GRU (Gated Recurrent Unit) and other methods. We suggest using several techniques to measure brain signals, such as functional Magnetic Resonance Imaging (fMRI) and magnetoencephalography (MEG). We also recommend other types of feature selection algorithms (such as hybrid cellular automation and Gray Wolf Optimizer) that can be used to study their effect on the model performance. 

## Figures and Tables

**Table 1:** Dataset description.

Description	Value
**Number of participants**	32
**Number of EEG channels**	32
**Number of videos (for each participant)**	40
**Frequency of sampling rate (before pre-processing)**	512 Hz
**Frequency of sampling rate (after pre-processing)**	128 Hz
**Description of labels**	
**Number of dataset label**	4 labels
**Names of labels**	Valence, liking, arousal, dominance
**Rating values for each label**	1 to 9
**Number of data for each label**	1280
**Data description**	
**Data numbers for each participant**	40 videos × 40 channels × 8064 data (numeric data).

**Table 2:** Features extracted from EEG signals.

Feature Type	Feature Name	Number of Features
Statistical features	Mean, Kurtosis, Skewness, Variance, Standard deviation, Minimum, Maximum, Median	8 features
Wavelet features	Wavelet Packet Decomposition Low Pass Filtering High Pass Filtering	20 features
Other features	Hurst exponent	40 features
Total number for feature extraction		68 features

**Table 3:** The hyperparameters’ values for BiLSTM.

No.	Parameter	Values
1	Learning rate	0.01
2	Optimization	ADAM
3	Maximum number of epochs	35
4	Minibatch Size	80
5	Hidden units	100
6	Gradient Threshold	1
7	Hidden layers	5 × 1 Layers
8	Execution Environment	Auto
9	Sequence Length	‘longest’
10	Shuffle	Once
11	Activation function	sigmoid

**Table 4:** Valence label results.

Class Name	Valence
**True Positive (TP)**	561
**True Negative (TN)**	712
**False Positive (FP)**	2
**False Negative (FN)**	5
**Accuracy**	99.4531
**Precision**	99.69
**Recall**	99.24
**F-score**	99.46

**Table 5:** Arousal label results.

Class Name	Arousal
**True Positive (TP)**	510
**True Negative (TN)**	730
**False Positive (FP)**	14
**False Negative (FN)**	26
**Accuracy**	96.8750
**Precision**	97.32
**Recall**	95.14
**F-score**	96.22

**Table 6:** Liking label results.

Class Name	Liking
**True Positive (TP)**	241
**True Negative (TN)**	855
**False Positive (FP)**	1
**False Negative (FN)**	3
**Accuracy**	99.6875
**Precision**	99.59
**Recall**	98.77
**F-score**	99.18

**Table 7:** Statistical test after feature selection.

Sample	Label	Q-Statistic	*p*-Value	Inference
The statistical test when comparing the model before and after features selection (BGWO)	Valence	3.531	0.0484	*p* < 0.05
	Arousal	3.827	0.0517	0.0517
	Liking	0.0425	0.0425	*p* < 0.05

**Table 8:** Comparison between our classification result and the results of previous works.

Accuracy				
Ref.	Classifier	Valence	Arousal	Liking
Yin al et al. [18]	GCNN & LSTM	90.45%	90.60%	-
Seeja et al. [22]	DNN	62%	63%	-
Numao et al. [25]	MLP	64.1%	73%	-
Girardi et al. [28]	SVM	56%	60.4%	-
Wabil et al. [4]	DNN	82%	-	-
Liu et al. [59]	Random forest	74.3%	77.2%	-
Akan et al. [60]	ANN	72.7%	75%	-
Yang et al. [61]	CNN	90.01 %	90.65%	-
Salama et al. [62]	3D CNN	87.44%	88.49%	-
Xing et al. [57]	LSTM	81.10%	74.38%	-
Li et al. [58]	LSTM	76.67%	-	-
Alhagry et al. [17]	LSTM-RNN	85%	85.4%	87.9%
Proposed method	Stacked Bi-LSTM	99.45%	96.87 %	99.68%

**Table 9:** Results of the proposed method.

	Accuracy	Precision	Recall	F-Score
**Valence**	99.4531	99.69	99.24	99.46
**Arousal**	96.8750	97.32	95.14	96.22
**Liking**	99.68	99.59	98.77	99.18

## Data Availability

The DEAP dataset license was obtained from the dataset’s official website. The data that were used consist of numerical content taken from the dataset available to the research community. Informed consent was obtained from all subjects involved in the study. The entity responsible for the data collection is Queen Mary University of London. Link: https://www.eecs.qmul.ac.uk/mmv/datasets/deap/, (accessed on: 16 October 2020).

## References

[1] Craik A., He Y., Contreras-Vidal J.L. (2019). Deep learning for electroencephalogram (EEG) classification tasks: A review. J. Neural Eng..

[2] Teles A., Cagy M., Silva F., Endler M., Bastos V.H., Teixeira S. (2017). Using Brain-Computer Interface and Internet of Things to Improve Healthcare for Wheelchair Users. Elev. Int. Conf. Mob. Ubiquitous Comput. Syst. Serv. Technol..

[3] Xu G., Ren T., Chen Y., Che W. (2020). A One-Dimensional CNN-LSTM Model for Epileptic Seizure Recognition Using EEG Signal Analysis. Front. Neurosci..

[4] Al-Nafjan A., Hosny M., Al-Wabil A., Al-Ohali Y. (2017). Classification of Human Emotions from Electroencephalogram (EEG) Signal using Deep Neural Network. Int. J. Adv. Comput. Sci. Appl..

[5] Du G., Zhou W., Li C., Li D., Liu P.X. (2020). An Emotion Recognition Method for Game Evaluation Based on Electroencephalogram. IEEE Trans. Affect. Comput..

[6] Kheirkhah M., Brodoehl S., Leistritz L., Götz T., Baumbach P., Huonker R., Witte O.W., Volk G.F., Guntinas-Lichius O., Klingner C.M. (2020). Abnormal Emotional Processing and Emotional Experience in Patients with Peripheral Facial Nerve Paralysis: An MEG Study. Brain Sci..

[7] Kamnitsas K., Ledig C., Newcombe V., Simpson J.P., Kane A.D., Menon D.K., Rueckert D., Glocker B. (2017). Efficient multi-scale 3D CNN with fully connected CRF for accurate brain lesion segmentation. Med. Image Anal..

[8] Abd-Ellah M.K., Awad A.I., Khalaf A.A.M., Hamed H.F.A. (2018). Two-phase multi-model automatic brain tumour diagnosis system from magnetic resonance images using convolutional neural networks. EURASIP J. Image Video Process..

[9] Deniz C.M., Xiang S., Hallyburton R., Welbeck A., Babb J., Honig S., Cho K., Chang G. (2018). Segmentation of the Proximal Femur from MR Images using Deep Convolutional Neural Networks. Sci. Rep..

[10] Ravi D., Wong C., Lo B., Yang G.Z. Deep learning for human activity recognition: A resource efficient implementation on low-power devices. Proceedings of the 2016 IEEE 13th International Conference on Wearable and Implantable Body Sensor Networks (BSN).

[11] Abdullah S., Choudhury T. (2018). Sensing Technologies for Monitoring Serious Mental Illnesses. IEEE MultiMedia.

[12] Lupu R.G., Ungureanu F., Cimpanu C. Brain-computer interface: Challenges and research perspectives. Proceedings of the 2019 22nd International Conference on Control Systems and Computer Science (CSCS).

[13] Mohammadpour M., Hashemi S.M.R., Houshmand N. Classification of EEG-based emotion for BCI applications. Proceedings of the 2017 Artificial Intelligence and Robotics (IRANOPEN).

[14] Bin S.H. (2019). Emotion Recognition Using EEG Signal and Deep Learning Approach. Ph.D. Thesis.

[15] Alarcao S.M., Fonseca M.J. (2019). Emotions Recognition Using EEG Signals: A Survey. IEEE Trans. Affect. Comput..

[16] George F.P., Shaikat I.M., Hossain P.S.F., Parvez M.Z., Uddin J. (2019). Recognition of emotional states using EEG signals based on time-frequency analysis and SVM classifier. Int. J. Electr. Comput. Eng..

[17] Alhagry S., Aly A., Reda A. (2017). Emotion Recognition based on EEG using LSTM Recurrent Neural Network. Int. J. Adv. Comput. Sci. Appl..

[18] Yin Y., Zheng X., Hu B., Zhang Y., Cui X. (2020). EEG emotion recognition using fusion model of graph convolutional neural networks and LSTM. Appl. Soft Comput..

[19] Cimtay Y., Ekmekcioglu E. (2020). Investigating the Use of Pretrained Convolutional Neural Network on Cross-Subject and Cross-Dataset EEG Emotion Recognition. Sensors.

[20] Wei C., Chen L.-L., Song Z.-Z., Lou X.-G., Li D.-D. (2020). EEG-based emotion recognition using simple recurrent units network and ensemble learning. Biomed. Signal Process. Control.

[21] Chao H., Liu Y. (2020). Emotion Recognition From Multi-Channel EEG Signals by Exploiting the Deep Belief-Conditional Random Field Framework. IEEE Access.

[22] Pandey P., Seeja K. (2019). Subject independent emotion recognition from EEG using VMD and deep learning. J. King Saud Univ. Comput. Inf. Sci..

[23] Thejaswini S., Ravikumar K.M., Jhenkar L., Natraj A., Abhay K.K. (2019). Analysis of EEG based emotion detection for DEAP and SEED-IV databases using SVM 208 II. Lit. Rev..

[24] Mohammadi Z., Frounchi J., Amiri M. (2017). Wavelet-based emotion recognition system using EEG signal. Neural Comput. Appl..

[25] Thammasan N., Moriyama K., Fukui K.-I., Numao M. (2016). Familiarity effects in EEG-based emotion recognition. Brain Inform..

[26] Zhuang N., Zeng Y., Yang K., Zhang C., Tong L., Yan B. (2018). Investigating Patterns for Self-Induced Emotion Recognition from EEG Signals. Sensors.

[27] Wang K.Y., Ho Y.L., de Huang Y., Fang W.C. Design of Intelligent EEG System for Human Emotion Recognition with Convolutional Neural Network. Proceedings of the 2019 IEEE International Conference on Artificial Intelligence Circuits and Systems (AICAS).

[28] Girardi D., Lanubile F., Novielli N. Emotion detection using noninvasive low cost sensors. Proceedings of the 2017 Seventh International Conference on Affective Computing and Intelligent Interaction (ACII).

[29] Ozdemir M.A., Degirmenci M., Guren O., Akan A. EEG based emotional state estimation using 2-D deep learning technique. Proceedings of the 019 Medical Technologies Congress (TIPTEKNO).

[30] Lowenthal M.N., Lieberman D. (1994). DEAP: A Database for Emotion Analysis using Physiological Signals. Isr. J. Med. Sci..

[31] Subasi A. (2007). EEG signal classification using wavelet feature extraction and a mixture of expert model. Expert Syst. Appl..

[32] Karegar F.P., Fallah A., Rashidi S. ECG based human authentication with using Generalized Hurst Exponent. Proceedings of the 2017 Iranian Conference on Electrical Engineering (ICEE).

[33] Geng S., Zhou W., Yuan Q., Cai D., Zeng Y. (2011). EEG non-linear feature extraction using correlation dimension and Hurst exponent. Neurol. Res..

[34] SMadan S., Srivastava K., Sharmila A., Mahalakshmi P. (2017). A case study on Discrete Wavelet Transform based Hurst exponent for epilepsy detection. J. Med. Eng. Technol..

[35] Mohan A.T., Gaitonde D.V. (2018). A deep learning based approach to reduced order modeling for turbulent flow control using LSTM neural networks. arXiv.

[36] Thejaswini S., Kumar K.M.R., Rupali S., Abijith V. (2018). EEG based emotion recognition using wavelets and neural networks classifier. Cognitive Science and Artificial Intelligence.

[37] Palendeng M.E. (2011). Removing Noise from Electroencephalogram Signals for BIS Based Depth of Anaesthesia Monitors Master of Engineering Research (MENR). Ph.D. Thesis.

[38] Hashem Y., Takabi H., GhasemiGol M., Dantu R. Inside the Mind of the Insider: Towards Insider Threat Detection Using Psychophysiological Signals. Proceedings of the 7th ACM CCS International Workshop on Managing Insider Security Threats.

[39] Zhang Y., Liu B., Ji X., Huang D. (2016). Classification of EEG Signals Based on Autoregressive Model and Wavelet Packet Decomposition. Neural Process. Lett..

[40] Al Ghayab H.R., Li Y., Abdulla S., Diykh M., Wan X. (2016). Classification of epileptic EEG signals based on simple random sampling and sequential feature selection. Brain Inform..

[41] Rashid T.A., Abbas D., Turel Y.K. (2019). A multi hidden recurrent neural network with a modified grey wolf optimizer. PLoS ONE.

[42] Shon D., Im K., Park J.-H., Lim D.-S., Jang B., Kim J.-M. (2018). Emotional Stress State Detection Using Genetic Algorithm-Based Feature Selection on EEG Signals. Int. J. Environ. Res. Public Health.

[43] Mirjalili S. (2015). How effective is the Grey Wolf optimizer in training multi-layer perceptrons. Appl. Intell..

[44] Emary E., Zawbaa H.M., Hassanien A.E. (2016). Binary grey wolf optimization approaches for feature selection. Neurocomputing.

[45] Sánchez D., Melin P., Castillo O. (2017). A Grey Wolf Optimizer for Modular Granular Neural Networks for Human Recognition. Comput. Intell. Neurosci..

[46] Pan J., Jing B., Jiao X., Wang S. (2020). Analysis and Application of Grey Wolf Optimizer-Long Short-Term Memory. IEEE Access.

[47] Al-Tashi Q., Kadir S.J.A., Rais H.M., Mirjalili S., Alhussian H. (2019). Binary Optimization Using Hybrid Grey Wolf Optimization for Feature Selection. IEEE Access.

[48] Emary E., Zawbaa H.M., Grosan C. (2017). Experienced Gray Wolf Optimization Through Reinforcement Learning and Neural Networks. IEEE Trans. Neural Netw. Learn. Syst..

[49] Hu X., Yuan Q. Epileptic EEG Identification Based on Deep Bi-LSTM Network. Proceedings of the 2019 IEEE 11th International Conference on Advanced Infocomm Technology.

[50] Du X., Ma C., Zhang G., Li J., Lai Y.-K., Zhao G., Deng X., Liu Y.-J., Wang H. (2020). An Efficient LSTM Network for Emotion Recognition from Multichannel EEG Signals. IEEE Trans. Affect. Comput..

[51] Hochreiter S., Schmidhuber J. (1997). Long short-term memory. Neural Comput..

[52] Yin J., Deng Z., Ines A.V., Wu J., Rasu E. (2020). Forecast of short-term daily reference evapotranspiration under limited meteorological variables using a hybrid bi-directional long short-term memory model (Bi-LSTM). Agric. Water Manag..

[53] Nagabushanam P., George S.T., Radha S. (2020). EEG signal classification using LSTM and improved neural network algorithms. Soft Comput..

[54] Elgeldawi E., Sayed A., Galal A.R., Zaki A.M. (2021). Hyperparameter Tuning for Machine Learning Algorithms Used for Arabic Sentiment Analysis. Informatics.

[55] Kingma D.P., Ba J.L. Adam: A method for stochastic optimization. Proceedings of the 3rd International Conference on Learning Representations, ICLR 201.

[56] Kuppens P., Tuerlinckx F., Russell J.A., Barrett L.F. (2013). The relation between valence and arousal in subjective experience. Psychol. Bull..

[57] Xing X., Li Z., Xu T., Shu L., Hu B., Xu X. (2019). SAE+LSTM: A New Framework for Emotion Recognition From Multi-Channel EEG. Front. Neurorobot..

[58] Li Z., Tian X., Shu L., Xu X., Hu B. (2018). Emotion recognition from EEG using RASM and LSTM. Commun. Comput. Inf. Sci..

[59] Liu J., Meng H., Li M., Zhang F., Qin R., Nandi A.K. (2018). Emotion detection from EEG recordings based on supervised and unsupervised dimension reduction. Concurr. Comput..

[60] Mert A., Akan A. (2018). Emotion recognition from EEG signals by using multivariate empirical mode decomposition. Pattern Anal. Appl..

[61] Yang H., Han J., Min K. (2019). A Multi-Column CNN Model for Emotion Recognition from EEG Signals. Sensors.

[62] Salama E.S., El-Khoribi R.A., Shoman M.E., Wahby M.A. (2018). EEG-Based Emotion Recognition using 3D Convolutional Neural Networks. Int. J. Adv. Comput. Sci. Appl..

